# Comparison of healing time and the histopathology of bone formation following tooth extraction using freeze-dried bone allograft:A randomized controlled clinical trial

**DOI:** 10.34172/japid.2022.020

**Published:** 2022-10-29

**Authors:** Omid Moghaddas, Nima Naddafpour, Sareh Farhadi, Peyman Nikookar, Sephora Khandan

**Affiliations:** ^1^Department of Periodontology, Faculty of Dentistry, Tehran Islamic Azad Medical Sciences University, Tehran, Iran; ^2^Department of Oral and Maxillofacial Pathology, Faculty of Dentistry, Tehran Islamic Azad Medical Sciences University, Tehran, Iran; ^3^General Practitioner, Private Practice, Tehran, Iran; ^4^Dental Branch, Tehran Medical Science, Islamic Azad University, Tehran, Iran

**Keywords:** Allografts, alveolar ridge augmentation, dental implant, bone resorption, bone graft, tooth extraction

## Abstract

**Background.** A decrease in the width and height of the alveolar ridge is inevitable following tooth extraction. This study aimed to histologically evaluate the amount of newly formed bone after using a freeze-dried bone allograft (FDBA) at two different intervals in the tooth socket grafting.

**Methods.** Forty patients were selected, who required a single-rooted tooth extraction and were candidates for implant placement, with no indication for an immediate implant. Extraction sockets were preserved using a cortical FDBA allograft in two regeneration interval groups: 3 months (group A) and 4 months (group B). At the time of implant placement, a bone sample was collected from each grafted socket. Histomorphometric analyses were performed to determine the percentage of newly formed bone and the residual graft material. Changes in histological indices, i.e., inflammation rate, percentage of ossification, and the amount of remaining biomaterial, were evaluated.

**Results.** There were no significant differences in the amount of newly formed bone and residual graft material between the two groups. In general, the average of new bone formation and remaining graft particles in groups A and B was: %33.89 and %12.59 vs. %39.83 and %14.07, respectively.

**Conclusion.** Bone parameters in group A were better compared to group B. However, due to the lack of significant differences in the results, it is suggested that implant placement in grafted sockets with mineralized allografts be expedited.

## Introduction

 The extraction socket dimensional changes might arouse serious concerns, prompting clinicians to perform reconstructive treatments to increase bone volume before implant placement.^1^ Approximately 0.34‒7.7 mm of resorption in the ridge width and 0.2‒3.25 mm of reduction in height occurs 6–12 months after tooth extraction,^2^ which is the best time to preserve tooth socket dimensions.^3^ The ridge preservation methods prevent 40–60% of alveolar bone atrophy following tooth extraction, which usually occurs 2–3 months after tooth extraction, and resorption continues at a rate of 0.25–0.5% per year.^4^ The use of graft materials to repair bone lesions or increase the width or height of atrophic alveolar ridges has been evaluated by several experimental studies, the first of which was conducted by Boyne^5^ in 1970 and is still cited in recent years due to its high success. Today, due to the increasing demand for implant treatments, various materials and techniques have been proposed to preserve the extraction socket dimensions, including allografts and alloplasts and xenograft particles.^6^ In severe cases of resorptive changes in the size of the alveolar ridge, it is difficult to place implants,^7^ and complex bone graft treatments are required.^8^ Although bone preservation supports fixed and removable prostheses, a successful osseointegration ensures the esthetic outcomes of final dental implant restorations.^3^ For years, the gold standard for bone grafting has been the use of autogenous bone from an intraoral source. Research on suitable bone grafting materials has increased in recent years due to the limitations of autografts in some patients, the need for surgery at the donor site, and the limitations of available bone volume.^9^ Allografts, including FDBA (freeze-dried bone allograft) and DFDBA (demineralized freeze-dried bone allograft), have been successful in many studies, with effective results in alveolar ridge preservation, minimizing ridge resorption following tooth extraction.^10,11^

 Since limited studies are available on the effect of ridge dimension preservation techniques after tooth extraction,^12,13^ with most being radiographic examinations and on animal models, this study aimed to histologically compare the FDBA graft material (absorbs with a slower rate compared to DFDBA) with natural socket healing in terms of bone quantity and quality for implant placement at different time intervals.

 Due to the long intervention period for tooth socket regeneration and due to the inconsistencies in various studies^10,14^ regarding the time required for proper bone formation (2, 3, and 4 months), the question is: “Is it possible to achieve the same success rate in bone formation in a shorter period (3 months) instead of 4 months?” Furthermore, histological studies have shown significantly different rates of bone formation at 2- and 4-month intervals.^14^ Therefore, due to the importance of time, this study evaluated the effect of FDBA material at 3- and 4-month intervals on the extraction socket bone formation.

## Methods

 This randomized controlled clinical trial (before-after) was approved under the ethical codes IR.IAU.DENTAL.REC.1396.12 and IRCTID: IRCT20170419033535N4.

 All the patients were selected from the Periodontology and Implant Department of the Faculty of Dentistry, Islamic Azad University, Tehran Branch, who required a single-rooted tooth extraction and did not have an indication for immediate implant placement. Exclusion criteria were infectious and systemic or local active diseases, known medical and pharmacological status altering the soft tissue and bone repair (uncontrolled or poorly controlled diabetes mellitus, bisphosphonates, and immunosuppressive drugs), pregnancy, short-rooted or malpositioned teeth, in which core biopsy would result in the involvement of the bony walls along the socket wall. After explaining the aim of this study to the 40 participants, informed consent was signed by the patients. Diagnostic procedures included radiographic evaluation, impression taking, preparing study casts, and clinical examination to evaluate the extraction site. After preparing the study casts, the stent was prepared as a fixed reference to determine the exact location of sampling from the extraction socket. Finally, the cortical FDBA graft material with 500‒1000-µm particles (Kish Tissue Regeneration Corporation, Iran) was used to graft the extraction socket.

###  Surgical procedure

**Figure 1:**
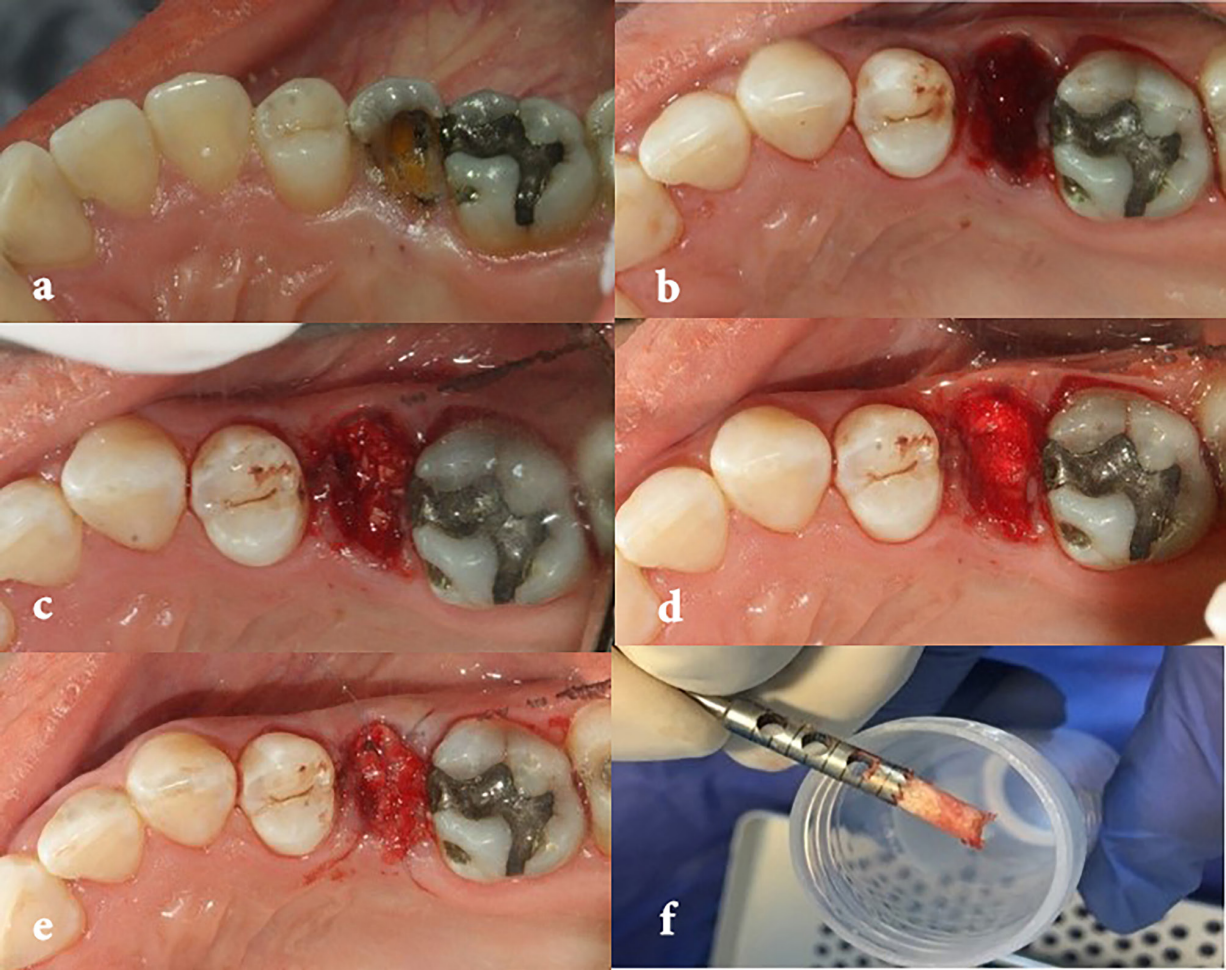
(a) tooth with a hopeless prognosis. (b) Atraumatic tooth extraction. (c) Placing the graft powder within the extraction socket. (d) Collagen coating on the socket. (e) Tension-free suturing the area, occlusal view. (f) Bone sampling with a trephine bur with an inner diameter of 2 mm and an outer diameter of 3 mm.

 surgery, each patient was randomly assigned to treatment groups using opaque envelopes (Figure 1). Following anesthesia of the patients with lidocaine with 1:80000 Before epinephrine, atraumatic extraction of the teeth, and debridement and complete rinsing, the presence or absence of dehiscence and the number of bony walls were recorded. Williams probe was used to ensure the presence of mesial, buccal, distal, and lingual bone walls through sounding. The graft was hydrated using sterile saline for 10 minutes and then placed in extracted tooth socket so that the socket was not overfilled. Extraction sockets were sealed with the collagen sponge (Ateloplug/Korea), and the area was sutured with 5-0 nylon cross mattress suture.

###  Post-surgical procedure

 Each patient was given 500 mg of amoxicillin/tid for seven days and 0.12% chlorhexidine mouthwash twice daily for 30 seconds for four weeks to eliminate microbial plaque. If the patient was allergic to penicillin, the patient was given 100 mg of doxycycline once a day for seven days. Postoperative pain was controlled with NSAIDs and opioid analgesics. Each patient was referred for a secondary surgical visit at the appointed time. To perform a core biopsy, a trephine bur with an inner diameter of 2 mm and an external diameter of 3 mm was used, and sampling was performed at a depth of at least 8 mm using a measuring stop. The bony samples were placed in a 10% neutral formalin buffer solution.

###  Examiner blindness

 At the first appointment, each patient was given a code, and in the second phase, biopsies were sent to the laboratory with the assigned code. The examiner evaluated the results based on the codes; therefore, he was unaware of the treatment groups.

###  Analysis and histological processes 

 Briefly, core biopsies were collected using a trephine bur and placed directly in the 10% neutral formalin buffer. The cores were decalcified, dehydrated, and embedded in paraffin. Then 4-µm-thick sections were prepared for histomorphometric examinations. Finally, the tissue was stained by conventional hematoxylin staining methods.^14^

**Figure 2:**
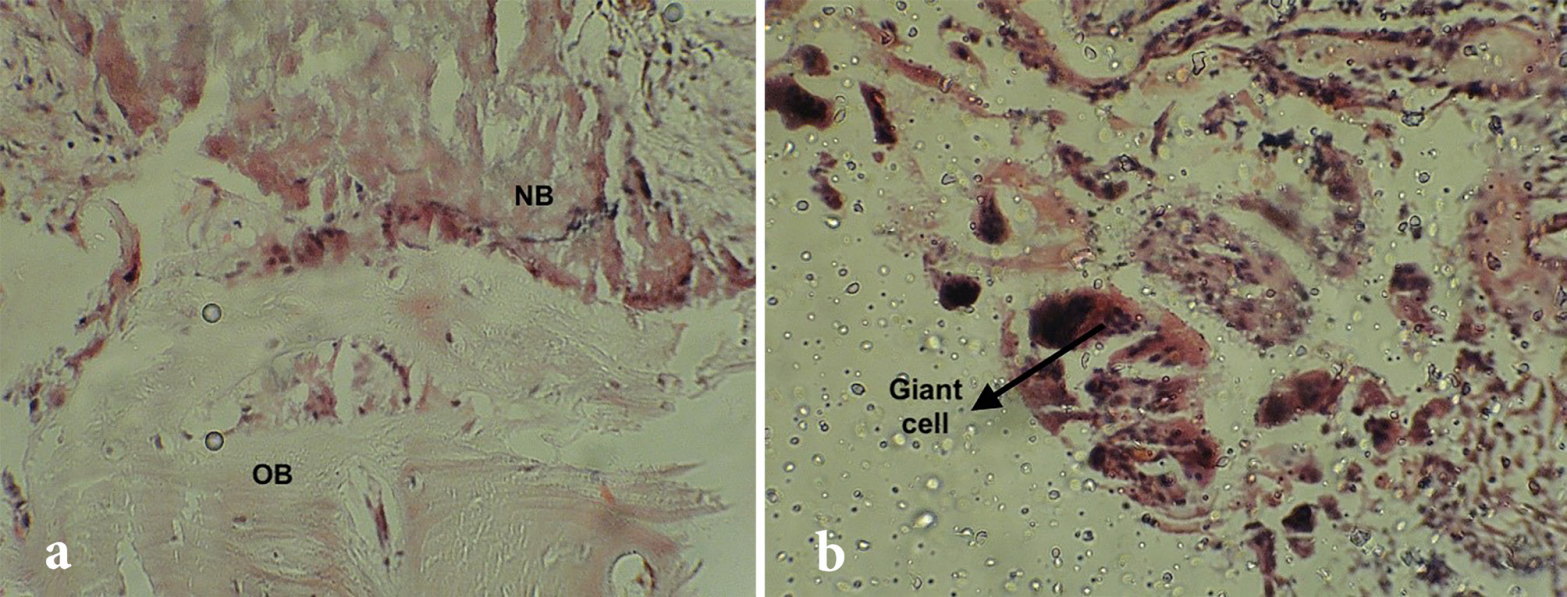
(a) and (b) Histological view of the samples. (a) Amount of new bone formation (NB) and old bone (OB). (b) Inflammation.

 The stained sections were examined by an oral pathologist to determine the percentage of viable bone, the amount of residual biomaterial, and inflammation at ×100 magnification of a Nikon YS-100 light microscope with a graduated lens (Figure 2).^15^

###  Statistical analysis 

 The changes in histological indices, i.e., inflammation rate, bone formation percentage, and the amount of remaining biomaterial, at the two time intervals were evaluated with the Mann-Whitney U test.

## Result

 This study aimed to evaluate the histological results of FDBA (Ceno Bone) allograft in preventing tooth socket resorption after extraction of 40 single-rooted teeth. In this study, half of the cases were sampled 3 months and the other 4 months after socket grafting. Fifty-two teeth were selected for the study, and 12 samples were excluded due to the patients’ lack of cooperation. Forty participants completed the study, including 38 males and 2 females. The mean ages of groups A and B were 53.1 and 54.5 years, respectively. Twenty samples were recalled after 3 months and twenty samples after 4 months. After the surgical procedure, the desired samples were prepared and sent to the pathology laboratory to evaluate the effect of the material. The final results of 40 samples are presented in Table 1 and in detail in Tables 2 and 3. The results did not show a significant difference between the two groups regarding bone formation. There was also no significant difference in the amount of residual biomaterial and inflammatory cells. The ossification rates in groups A and B were 33.89±8.88% and 39.83±11.32%, respectively, which did not differ significantly. The amount of residual biomaterial in group A was 12.59±6.36%, with 14.07±5.71% in group B; the inflammation rate was reported to be 1.55±0.94 (group A) and 1.04±0.82 (group B), with no significant difference.

**Table 1:** Inflammation rate, percentage of remaining biomaterial, and inflammation rate over time

Interval	Inflammation	Bone formation percentage	Residual biomaterial percentage
Group B	1.55±0.94*	39.83±11.32	14.07±5.71
Group A	1.04±0.82	33.89±8.88	12.59±6.36
Test result	P=0.642(NS)†	P=0.091(NS)	P=0.330(NS)

*Mean and SD †Not significant

**Table 2:** Patients’ characteristics, Histomorphometric evaluations in group B

**Sample no**	**Tooth no**	**Age**	**Gender**	**Inflammation Grade**	**Amount of Bone Formed**	**Residual Biomaterial**
**B11**	21	55	Male	1	53.9	9.2
**B12**	34	55	Male	2	49.1	8.3
**B13**	25	70	Male	1	40.8	20.1
**B14**	12	70	Male	0	39.9	21.4
**B15**	24	70	Male	2	35.8	17.3
**B16**	25	70	Male	3	61.3	22.2
**B17**	34	70	Male	2	49.1	29.1
**B18**	35	70	Male	0	33.8	11.2
**B23**	43	41	Male	1	51.2	15.3
**B24**	11	41	Male	2	39.4	13.7
**B25**	44	41	Male	1	38.8	12.9
**B29**	15	45	Male	1	29.3	8.8
**B31**	11	45	Male	2	37.7	11
**B32**	42	65	Male	1	33.6	12.1
**B35**	11	39	Male	1	32.4	9.1
**B36**	12	39	Male	1	52.3	8.2
**B37**	22	39	Male	2	50	7.3
**B38**	21	39	Male	3	29.6	11.9
**B39**	31	63	Male	1	17.3	15.4
**B40**	32	63	Male	1	21.4	16.9

**Table 3:** Patients’ characteristics, Histomorphometric evaluations in group A

**Sample no**	**Tooth no**	**Age**	**Gender**	**Inflammation Grade**	**Amount of Bone Formed**	**Residual Biomaterial**
**B1**	11	49	Male	3	38.9	14.1
**B2**	44	49	Male	0	34.4	13.5
**B3**	22	49	Male	1	44.6	28.1
**B4**	34	40	Female	1	35.6	29.9
**B5**	32	78	Male	1	48.3	13.4
**B6**	22	78	Male	3	18.9	10.8
**B7**	44	40	Female	3	31.4	9
**B8**	42	78	Male	1	21.1	4.1
**B9**	15	78	Male	1	32.5	6.1
**B10**	31	78	Male	2	31.8	12.9
**B19**	24	37	Male	1	24.6	7.3
**B20**	25	37	Male	0	43.9	6.4
**B21**	14	37	Male	3	20.7	14.3
**B22**	15	37	Male	1	24.3	15
**B26**	13	45	Male	2	43.1	11.3
**B27**	12	45	Male	1	27.8	10.6
**B28**	25	45	Male	2	36.5	12.7
**B30**	21	45	Male	2	34.1	9.3
**B33**	43	65	Male	1	39.9	12.4
**B34**	33	52	Male	2	45.4	10.6

## Discussion

 This study aimed to evaluate the effect of regeneration time on newly formed bone following ridge preservation with FDBA (Ceno Bone Kish Tissue Regeneration Corporation, Iran) particles 3 and 4 months after tooth extraction to minimize the intervening variable effect and allow direct and accurate comparison of the amount of newly formed bone in groups A and B.^14^ The same graft material was used in both groups’ extraction sockets with a minimum length of 10 mm and root angulation similar to the desired position of the final implant. To eliminate the misalignment of native bone in performing a biopsy, a detailed histological examination and accurate reporting of the percentage of bone formation were carried out, and an acrylic stent was used to determine the exact location of the tooth for biopsy and bone sampling.

 All sites showed newly formed bone. There was no significant difference in the amount of newly formed bone between 3- and 4-month follow-ups (33.89% vs. 39.83%).

 In Wood and Mealey’s^10^ study, after 19 weeks, the FDBA group showed 24.63% vital bone formation. However, it should be noted that in this study, a stent was not used to determine the exact location of the extraction socket, and it was possible to make a mistake in finding the exact location of the biopsy for sampling the native bone.

 The results showed that the rate of ossification in 3 months was about 33.89%, consistent with a study by Sarkarat et al,^16^ in which there was 36.65% ossification of FDBA Ceno Bone allograft after three months.

 Wang and Tsao^17^ evaluated alveolar ridge augmentation with mineralized human allograft in 7 areas with 16‒20-week follow-ups, reporting that the amount of newly formed bone was 68%.

 In the study by Trombelli et al,^18^ the amount of woven bone formation in a 6-month follow-up was reported at 32.36%. All the available studies suggested that ridge preservation techniques with human mineralized allografts can lead to new bone formation in the extracted tooth socket.

 Using different graft materials and ridge preservation techniques, different new bone growth rates, residual graft material percentages, and connective tissue formation have been reported. This variation can be influenced by various factors, including the status of pre-extraction periodontal diseases, single and multi-rooted teeth, the size of the extracted tooth, the presence or absence of bone fenestration or dehiscence, trauma during tooth extraction, structural damage to periodontium before tooth extraction, the angle of the core biopsy and the tooth angulation.^19^

 In a study by Cammack et al,^20^ the mean bone formation percentage in the FDBA group at 6–36 months was 41.89%. Such a discrepancy might be attributed to the difference in the intervals to collect biopsies from the samples, and it was not stated when and how many samples had complete absorption. The number of residual particles in the FDBA group was 9.86±7.69 in the ridge augmentation sites and 17.86±9.56% for sinus augmentation at 6–36-month intervals, which was lower than the residual graft rate in the present study.

 Beck and Mealey’s^19^ study showed no difference in the percentage of newly formed bone and residual graft particles 3 and 6 months after grafting the sockets (45.8% vs. 45%). The particle size in their study was 250‒1000 µm, which was in the particle size range in the present study. Stents were not used. The allograft material used was cancellous, whereas the material used in the present study was cortico-cancellous. However, in the present study, the amount of bone formation after 3 months was 33.89%, much lower than that in Beck and Mealey’s study.^19^

 Borg and Mealey^11^ showed higher bone formation in the mineralized/demineralized compound at a ratio of 70:30 compared to FDBA alone, suggesting a possible osteoinductive effect of DFDBA.

 In Eskow and Mealey’s^21^ study, after 18 weeks of follow-up, new bone formation rates were 12.98% and 16.08% in the FDBA cancellous group and in the FDBA cortical group, respectively, with no significant difference. In this study, the residual graft material rates were 38.28% in the cortical group and 19.94% in the cancellous group, indicating a significant difference compared with the present study. It shows that the type of graft material influences the residual graft and new bone formation rate more than time. However, comparative research shows that the entire cancellous graft resorbs in two years, whereas a portion of the cortical graft remains. In the Eskow and Mealey’s^21^ study, sampling was performed only once, with no data on bone turnover and residual graft shrinkage over time.

 The present study showed no significant difference between groups A and B. The rate of inflammation in group A was 1.55±0.94, with 1.4±0.82 in group B, indicating mild inflammation. According to the variables’ table in group A, 10% of the samples showed no inflammatory cells (grade 0), 47.5% of the samples showed mild inflammation (grade I), with 27.5% local inflammation (grade II), and 15% local inflammation (grade III).

 Amooian et al^22^ evaluated the clinical, histological, and histomorphometric results of bone strip allograft (Ceno Bone) in the horizontal alveolar ridge augmentation, reporting that the rate of inflammation in most samples (85.7%) was grade I. No external reaction of the samples was observed. The bone was vital in all the samples. The percentage of bone formation was 58.43±26.42%, and the amount of residual biomaterial was 4.07±2.44%. This material has good porosity for the penetration of blood vessels and nutrients from the surrounding tissues, and it has a surface that allows it to adhere to and express the ossification phenotype.

 In the present study, mature lamellar bone formation was observed in both groups. Signs of newly formed bone, including new vascularization and osteocyte cells inside the lacunae, were seen in all the samples. Bone remodeling was detected by osteoclasts and reversal lines. The amount of residual biomaterial and connective tissue varies in different studies and depends on various parameters, including the surgical procedure, the type of graft material to fill the extraction socket, and the recovery period. Findings and parameters of connective tissue healing and residual graft are consistent with other studies on allograft composition.^23^

 In this study, all the surgeries were performed without flap retraction because raising the periosteum from the buccal bone to create a mucoperiosteal flap can reduce blood flow to the exposed bone, activate osteoclasts, and eventually lead to bone resorption. This minimally invasive approach is associated with higher patient satisfaction, shorter surgical time, and, most importantly, the absence of mucogingival junction displacement, which helps better keratinized soft tissue formation in the affected areas.^24^ In this study, the grafted sockets healed favorably.

 In histomorphometric studies on microscopic sections, despite the many advantages, the presence of a two-dimensional image of 3D space leads to limitations in the study and interpretation of histological sections of reconstructed areas of bone.^25^ Therefore, in addition to the influence of biological factors on the thickness of bony trabeculae, technical issues such as the preparation of sections relative to the longitudinal axis of the defect (vertical or transverse) are also quite effective in the obtained microscopic view and can explain the differences between the results of studies. It is worth mentioning that the method of obtaining bone samples in human studies is different from each others, which can influence the interpretation of the results.^26^

## Conclusion

 Within the limitations of the present study, using the cortical type of FDBA in extraction sockets showed better bone parameters in 4 months compared to 3 months, although the differences were not significant. Therefore, in cases of single-rooted tooth extraction and needing delayed implant placement, the implant can be placed in a shorter period.

## Acknowledgments

 None.

## Competing interests

 No conflicts of interest are associated with this publication.

## Authors’ contributions

 OM conceived the presented idea, prepared the study, and scientifically edited the manuscript. OM and NN contributed to methods and materials. OM, NN, and PN managed clinical procedures. SF evaluated histopathology samples. PN collected the data. SKH analyzed and interpreted the data and prepared and reviewed the manuscript with support from OM.

## Funding

 This study was self-funded, and there has been no significant financial support for this work that could have influenced its outcomes.

## Availability of data

 Detailed information is available.

## Ethics approval

 Islamic Azad University, Dental. Branch Tehran, Iran. Research Ethics Committees Certificate Approval ID: IR.IAU.DENTAL.REC.1396.12

 Iranian Registry of Clinical Trials IRCTID: IRCT20170419033535N4.
